# Risk of newly detected infections and cervical abnormalities in adult women seropositive or seronegative for naturally acquired HPV‐16/18 antibodies

**DOI:** 10.1002/cam4.1879

**Published:** 2019-07-05

**Authors:** Dominique Rosillon, Laurence Baril, Maria Rowena Del Rosario‐Raymundo, Cosette Marie Wheeler, Susan Rachel Skinner, Suzanne Marie Garland, Jorge Salmeron, Eduardo Lazcano‐Ponce, Carlos Santiago Vallejos, Tanya Stoney, Bram ter Harmsel, Timothy Yong Kuei Lim, Swee Chong Quek, Galina Minkina, Shelly Ann McNeil, Celine Bouchard, Kah Leng Fong, Deborah Money, Arunachalam Ilancheran, Alevtina Savicheva, Margaret Cruickshank, Archana Chatterjee, Alison Fiander, Mark Martens, Marie Cecile Bozonnat, Frank Struyf, Gary Dubin, Xavier Castellsagué

**Affiliations:** ^1^ GSK Wavre Belgium; ^2^ Department of Obstetrics and Gynecology San Pablo Colleges Medical Center San Pablo City The Philippines; ^3^ University of New Mexico Health Sciences Center Albuquerque New Mexico; ^4^ Vaccines Trials Group Telethon Kids Institute Perth Western Australia Australia; ^5^ Sydney University Discipline of Paediatrics and Child Health, Children's Hospital Westmead Sydney New South Wales Australia; ^6^ The Royal Women's Hospital, The Royal Children's Hospital, Murdoch Childrens Research Institute University of Melbourne Parkville Victoria Australia; ^7^ Instituto Mexicano del Seguro Social Morelos Mexico; ^8^ National Institute of Public Health Cuernavaca Mexico; ^9^ Departamento de Oncología Médica Oncosalud‐AUNA Lima Peru; ^10^ Telethon Kids Institute University of Western Australia Perth Western Australia Australia; ^11^ Department of Gynecology Roosevelt Kliniek, Leiden Delft The Netherlands; ^12^ KK Hospital Singapore City Singapore; ^13^ ASC Clinic for Women Gleneagles Hospital Singapore City Singapore; ^14^ City Clinical Hospital Moscow Russia; ^15^ Canadian Center for Vaccinology, IWK Health Centre and Nova Scotia Health Authority Dalhousie University Halifax Nova Scotia Canada; ^16^ Clinique de Recherche en Santé des Femmes Québec City Québec Canada; ^17^ Singapore General Hospital Singapore City Singapore; ^18^ The Women's Health Research Institute University of British Columbia Vancouver British Columbia Canada; ^19^ Division of Gynaecologic Oncology, Department of Obstetrics and Gynaecology National University Hospital Singapore City Singapore; ^20^ Laboratory of Microbiology DO Ott Research Institute of Obstetrics, Gynaecology and Reproductology St. Petersburg Russia; ^21^ Department of Obstetrics and Gynaecology, Aberdeen Maternity Hospital NHS Grampian Scotland, UK; ^22^ Department of Pediatrics University of South Dakota Sanford School of Medicine/Sanford Children's Specialty Clinic Sioux Falls South Dakota; ^23^ Leading Safe Choices Programme Royal College of Obstetricians and Gynaecologists London UK; ^24^ Reading Hospital Pennsylvania; ^25^ 4Clinics Paris France; ^26^ GSK King of Prussia Pennsylvania; ^27^ Institut Català d'Oncologia (ICO) IDIBELL, CIBER‐ESP, L'Hospitalet de Llobregat Catalonia Spain

**Keywords:** human papillomavirus infection, naturally acquired antibodies, redetection or reactivation of HPV infection, cervical abnormality, risk reduction

## Abstract

**Background:**

Infections with human papillomavirus (HPV) types 16 and 18 account for ~70% of invasive cervical cancers but the degree of protection from naturally acquired anti‐HPV antibodies is uncertain. We examined the risk of HPV infections as defined by HPV DNA detection and cervical abnormalities among women >25 years in the Human Papilloma VIrus Vaccine Immunogenicity ANd Efficacy trial's (VIVIANE, NCT00294047) control arm.

**Methods:**

Serum anti‐HPV‐16/18 antibodies were determined at baseline and every 12 months in baseline DNA‐negative women (N = 2687 for HPV‐16 and 2705 for HPV‐18) by enzyme‐linked immunosorbent assay (ELISA) from blood samples. HPV infections were identified by polymerase chain reaction (PCR) every 6‐months, and cervical abnormalities were confirmed by cytology every 12 months. Data were collected over a 7‐year period. The association between the risk of type‐specific infection and cervical abnormalities and serostatus was assessed using Cox proportional hazard models.

**Results:**

Risk of newly detected HPV‐16‐associated 6‐month persistent infections (PI) (hazard ratio [HR] = 0.56 [95%CI:0.32; 0.99]) and atypical squamous cells of undetermined significance (ASC‐US+) (HR = 0.28 [0.12; 0.67]) were significantly lower in baseline seropositive vs baseline seronegative women. HPV‐16‐associated incident infections (HR = 0.81 [0.56; 1.16]) and 12‐month PI (HR = 0.53 [0.24; 1.16]) showed the same trend. A similar trend of lower risk was observed in HPV‐18‐seropositive vs ‐seronegative women (HR = 0.95 [0.59; 1.51] for IIs, HR = 0.43 [0.16; 1.13] for 6‐month PIs, HR = 0.31 [0.07; 1.36] for 12‐month PIs, and HR = 0.61 [0.23; 1.61] for ASC‐US+).

**Conclusions:**

Naturally acquired anti‐HPV‐16 antibodies were associated with a decreased risk of subsequent infection and cervical abnormalities in women >25 years. This possible protection was lower than that previously reported in 15‐ to 25‐year‐old women.

## 1 BACKGROUND

Infections with human papillomavirus (HPV) types 16 and 18 are responsible for approximately 70% of invasive cervical cancers.^1^ While most infections clear on their own, some develop into precancerous lesions and cervical cancer.

Previous studies have shown that many women with incident HPV‐16 or HPV‐18 infections develop serum antibodies of the corresponding type of HPV.^2^, ^3^, ^4^, ^5^, ^6^, ^7^, ^8^ These naturally acquired antibodies can remain detectable for at least 4‐5 years after the initial infection.^9^ Whether or not these naturally acquired antibodies protect against future infection remains debatable.^10^, ^11^, ^12^, ^13^, ^14^, ^15^, ^16^, ^17^, ^18^

Risk of incident HPV infections in adult women is positively associated with new sexual partners and with the lifetime number of sexual partners.^19^, ^20^ In older women, both new viral acquisition and intermittent detections of HPV from past HPV exposures are likely to account for what has been classified as apparent new HPV infections. In women 30‐50 years of age, factors associated with repeat HPV detection have been shown to be comparable in short‐term and longer‐term studies, suggesting association between short‐term repeat detection and long‐term persistence.^21^ As incident HPV detection is negatively associated with viral load as well as with repeat detection, this suggests that actual new acquisition of HPV is less common than reactivation or intermittent persistence.

The role of naturally acquired antibodies in the prevention of new infections and cervical abnormalities can be explored in the control arms of large HPV vaccine trials. A correlation between naturally acquired antibodies to HPV‐16 (and to a lesser extent HPV‐18) and reduced risk of newly detected infection was demonstrated in younger women (15‐25 years) in the control arm of the PApilloma TRIal against Cancer In young Adults (PATRICIA; NCT00122681).^12^ Here, we examined the risk of “newly” detected HPV infections and cervical abnormalities among women >25 years in relation to naturally acquired HPV‐16/18 antibodies in the control arm of the VIVIANE during a 7‐year follow‐up period.^22^, ^23^

Our aim was to assess whether the risk factors for HPV infection differed between seropositive and seronegative women. We also analyzed risk factors stratified by baseline serostatus to mitigate the limitations in differentiating between new and reactivated infections.

## 2 METHODS

### 2.1 Study participants and procedures

Women aged >25 years were included in the control arm of the multinational, VIVIANE trial and were followed up for seven years. VIVIANE is the Human Papilloma Virus: Vaccine Immunogenicity and Efficacy trial. This is a phase 3 double‐blind, controlled vaccine trial based on age, cytology, region, and serostatus.^23^ The methodology of VIVIANE has been presented in detail elsewhere.^24^

**Figure 1:**
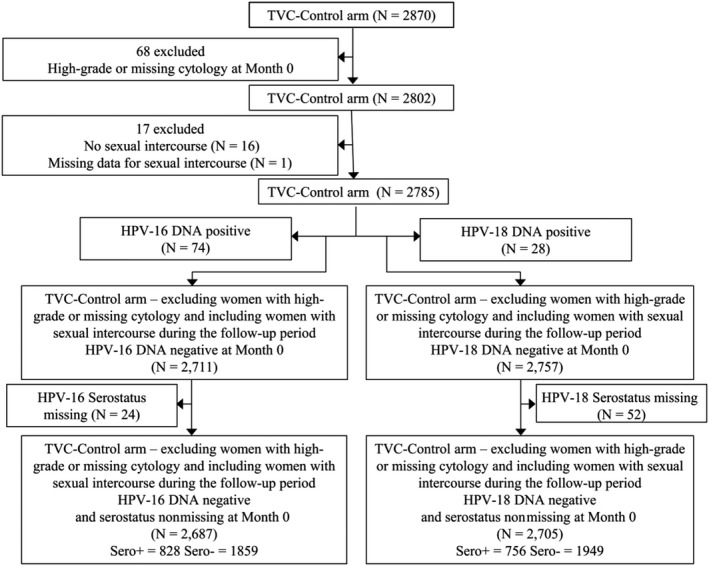
Flowcharts. HPV, human papillomavirus; TVC, total vaccinated cohort; N, number of women; Sero+, women seropositive for HPV‐16/18; Sero −, women seronegative for HPV‐16/18

Our analysis included women DNA‐negative for HPV‐16 and −18 at Month 0, with normal or low‐grade cytology (ie, negative or atypical squamous cells of undetermined significance [ASC‐US] or low‐grade squamous intraepithelial lesion [LSIL]) at Month 0, who had received at least one control vaccine dose (Al[OH]_3_) and who had sexual intercourse before or during the follow‐up (Figure 1).

Serum anti‐HPV‐16/18 antibodies were determined by enzyme‐linked immunosorbent assay (ELISA) from blood samples collected at baseline and every 12 months thereafter. Seropositivity was defined as an antibody level greater than or equal to the assay cutoff which was 8 ELISA units (EU)/mL for HPV‐16 and 7 EU/mL for HPV‐18.^25^

Liquid‐based cytology samples were tested for HPV using DNA typing PCR‐based assays every six months and cytopathological examinations every12 months.^25^ Information on known risk factors that predispose women to HPV cervical infection or recognized cofactors for cervical carcinogenesis was also collected through questionnaires. These data were collected at study entry and included demographic information, smoking habits, past and current sexual history, and reproductive status. In addition, data on participants' sexual behavior and use of contraception were collected every six months up to month 48.

Written informed consent was obtained from each woman before any study‐specific procedures were implemented. The protocol and other materials were approved by a national, regional, or investigational center Independent Ethics Committee or Institutional Review Board. The trial was conducted based on the Code of Ethics of the World Medical Association (Declaration of Helsinki).

The endpoints included in these analyses were (a) newly detected HPV‐16 and HPV‐18 incident infections, (b) 6‐ and 12‐month persistent infection (PI), ASC‐US+, and (c) histopathologically confirmed cervical intraepithelial neoplasia grade 1 or greater (CIN1+ and CIN2+). HPV‐16 and HPV‐18 serostatus were the main exposure variables.

### 2.2 Statistics

The analyses were performed on the total vaccinated cohort (TVC) of the control arm of the VIVIANE trial and included all women who received at least one control vaccine dose, who were DNA‐negative for HPV‐16 and HPV‐18 at Month 0, and who also had a normal or low‐grade cytology (ie, negative or ASC‐US or LSIL) at Month 0. All analyses were performed on women who had ever had sexual intercourse before study entry or during the follow‐up period.

Analyses were performed using SAS version 9.2. The incidence rate (IR) was calculated as the number of incident events divided by the total person‐time. Person‐years were calculated as the sum of the follow‐up for each participant expressed in years. The follow‐up period started on the day after first vaccination (control vaccine) and ended on the first occurrence of the endpoint or the last visit (whichever occurred first). The relationship between the exposure variables and the risk of newly detected infections or cervical abnormalities was assessed using Cox proportional hazard models. Univariate analyses were done to obtain unadjusted hazard ratios of the determinants of interest (not shown). For each endpoint, the following multivariable Cox models were performed including:

1. the type‐specific serostatus at baseline as a binary variable;
2. the type‐specific serostatus as a binary time‐dependent variable;
3. the antibody level as a time‐dependent continuous variable;
4. log‐transformed antibody level as a time‐dependent continuous variable.

For each endpoint, we included nine covariates in these models: region, age at inclusion, age at first sexual intercourse, marital status, smoking status at baseline, number of sexual partners during the past year, previous pregnancy, history of *Chlamydia trachomatis* infection, history of HPV infection/treatment or nonintact cervix. HPV‐associated infection or treatment was defined as two or more abnormal smears in sequence, an abnormal colposcopy or biopsy, or treatment of the cervix after abnormal smear or colposcopy findings. The histories of HPV infection/treatment were collected at baseline using medical history.

For ASC‐US+ only, previous type‐specific HPV infection was included as a time‐dependent variable since the presence of these cells indicates an active infection at a specific point in time. For CIN1+ and CIN2+ endpoints, no inferential analyses were performed due to the low number of cases. Also, analyses of determinants of interest were performed separately for the baseline seronegative and seropositive subjects to help determine whether newly detected infections were new or had been reactivated. The analysis is based on two assumptions: (a) An association between a latent reactivated infection and a known risk factor should be weaker than an association between a new infection and a known risk factor. (b) The reactivation of a PI should be more frequent in the baseline seropositive (representing presumed prior HPV infection exposure) subjects than in the baseline seronegative (representing presumed naïve, absent prior HPV infection exposure) subjects.

## 3 RESULTS

### 3.1 Study population

In total, 2687 and 2705 participants were included in the analysis of HPV‐16 and HPV‐18 endpoints, respectively (Figure 1). There was a difference of 3% between HPV‐16/18 by serostatus at baseline. Seroprevalence at enrollment was 31% (828/2687 seropositive women) for HPV‐16 and 28% (756/2705 seropositive women) for HPV‐18 (Table 1). This difference is entirely in agreement with the well‐known higher prevalence of 16 than 18 in HPV infections.

**Table 1:** Frequency distributions of exposure variables and risk factors at study entry ‐ TVC‐Control arm‐excluding high grade or missing cytology at Month 0 – Ever had sexual intercourse

		Overall (N = 2785)		Baseline HPV‐16 serostatus (N = 2687)				Baseline HPV‐18 serostatus (N = 2705)			
				Sero− (N = 1859)		Sero+ (N = 828)		Sero− (N = 1949)		Sero+ (N = 756)	
Exposure variables and Risk factors	Category	n	%	n	%	n	%	n	%	n	%
Marital status	Living or Lived with partner	2354	84.52	1629	87.63	659	79.59	1677	86.04	618	81.75
	Single	430	15.44	230	12.37	169	20.41	271	13.90	138	18.25
	Missing	1	0.04					1	0.05		
Number of pack years	[0; 0.5]	2016	72.39	1415	76.12	549	66.30	1451	74.45	514	67.99
	≥0.5	757	27.18	439	23.61	273	32.97	493	25.30	237	31.35
	Missing	12	0.43	5	0.27	6	0.72	5	0.26	5	0.66
Smoking status at baseline	No	2398	86.10	1639	88.17	688	83.09	1702	87.33	631	83.47
	Yes	386	13.86	220	11.83	140	16.91	246	12.62	125	16.53
	Missing	1	0.04	.		.		1	0.05	.	
Sexual history at study entry	No	5	0.18	4	0.22	1	0.12	4	0.21	1	0.13
	Yes	2779	99.78	1855	99.78	827	99.88	1944	99.74	755	99.87
	Missing	1	0.04	.		.		1	0.05	.	
History of HPV – Infection/treatment or not intact cervix	No	2431	87.29	1674	90.05	685	82.73	1725	88.51	636	84.13
	Yes	354	12.71	185	9.95	143	17.27	224	11.49	120	15.87
Age at first sexual intercourse (years)	<15	141	5.06	66	3.55	67	8.09	73	3.75	59	7.80
	15–17	899	32.28	507	27.27	354	42.75	557	28.58	309	40.87
	18–25	1561	56.05	1137	61.16	377	45.53	1173	60.18	354	46.83
	>26	177	6.36	146	7.85	27	3.26	141	7.23	32	4.23
	Missing	7	0.25	3	0.16	3	0.36	5	0.26	2	0.26
Number of lifetime sexual partners	0	5	0.18	4	0.22	1	0.12	4	0.21	1	0.13
	1	1068	38.35	876	47.12	174	21.01	864	44.33	183	24.21
	2–5	1017	36.52	666	35.83	310	37.44	703	36.07	286	37.83
	6–10	361	12.96	177	9.52	167	20.17	214	10.98	130	17.20
	11–15	146	5.24	68	3.66	69	8.33	79	4.05	60	7.94
	16–20	67	2.41	23	1.24	41	4.95	30	1.54	35	4.63
	>20	120	4.31	45	2.42	66	7.97	54	2.77	61	8.07
	Missing	1	0.04	.	.			1	0.05	.	
Number of sexual partners during the last year	0	301	10.81	195	10.49	96	11.59	214	10.98	85	11.24
	1	2219	79.68	1533	82.46	623	75.24	1585	81.32	574	75.93
	2‐3	230	8.26	115	6.19	95	11.47	132	6.77	83	10.98
	≥4	34	1.22	16	0.86	14	1.69	17	0.87	14	1.85
	Missing	1	0.04					1	0.05		
At least one previous pregnancy	No	439	15.76	277	14.90	138	16.67	299	15.34	119	15.74
	Yes	2345	84.20	1582	85.10	690	83.33	1649	84.61	637	84.26
	Missing	1	0.04					1	0.05		
*Chlamydia trachomatis*	No	2626	94.29	1794	96.50	741	89.49	1862	95.54	693	91.67
	Yes	133	4.78	56	3.01	72	8.70	70	3.59	55	7.28
	Missing	26	0.93	9	0.48	15	1.81	17	0.87	8	1.06
Contraception during lifetime^a^	No contraception	413	14.83	298	16.03	102	12.32	297	15.24	110	14.55
	Hormonal use for contraception or another indication	1802	24.85	1139	61.27	596	71.98	1220	62.60	515	68.12
	Intra‐Uterine Device	692	25.96	477	25.66	193	23.31	501	25.71	172	22.75
	Sterilized	723	35.30	498	26.79	204	24.64	522	26.78	183	24.21
Menopausal Status	Premenopausal	2448	87.90	1636	88.00	721	87.08	1717	88.10	657	86.90
	Perimenopausal	180	6.46	116	6.24	60	7.25	117	6.00	59	7.80
	Postmenopausal	142	5.10	93	5.00	46	5.56	101	5.18	39	5.16
	Missing	15	0.54	14	0.75	1	0.12	14	0.72	1	0.13

N = total Number of subjects with a given group.

a A subject can be included in more than one category.

Among those seropositive at enrollment, the geometric mean antibody concentration was 38.3 EU/mL (range: 8‐2527) and 23.3 EU/mL (range: 7‐725) for HPV‐16 and HPV‐18, respectively.

At enrollment, 45% of women were 26‐35 years old, 44% were 36‐45 years old, and 11% were ≥46 years old. Nearly all participants had been previously sexually active at the start of the study, except five who had their first sexual intercourse during the follow‐up. 56% had started sexual activity between 18 and 25 years (32% between 15 and 17), 80% had had one sexual partner during the previous year, and 84% had had a previous pregnancy. Moreover, 14% of women were current smokers, 5% were *C trachomatis*‐positive, and 87.8% were classified as pre‐menopausal, 6.5% as peri‐menopausal, 5.1% as post‐menopausal, while the status for the remaining 0.5% was missing.

### 3.2 Incidence rates of the endpoints

The IR per 100 person‐years of newly detected infections was 1.07 (95% confidence interval [CI]: 0.91‐1.25) for HPV‐16 and 0.64 (0.52‐0.78) for HPV‐18. For 6‐month PI, the IRs were 0.56 (0.44‐0.69) for HPV‐16 and 0.23 (0.16‐0.32) for HPV‐18. For 12‐month PI, these were 0.30 (0.22‐0.40) for HPV‐16 and 0.13 (0.08‐0.20) for HPV‐18.

The IRs for ASC‐US+ were 0.34 (0.26‐0.45) for HPV‐16 and 0.21 (0.14‐0.30) for HPV‐18.

During the seven years of follow‐up, 13 new HPV‐16 CIN1+ cases, 14 HPV‐18 CIN1+ cases, 8 HPV‐16 CIN2+ cases, and 9 HPV‐18 CIN2+ cases were detected.

### 3.3 Multivariable models

The multivariable Cox proportional hazard model, including the serostatus at baseline as a binary variable, showed that the risk of newly detected HPV‐16, 6‐month PI and ASC‐US+ was statistically significantly lower in seropositive vs seronegative women (hazard ratio [HR] = 0.56 [0.32‐0.99; *P* = 0.04] and 0.28 [0.12‐0.67; *P* = 0.004], respectively; Table 2). Analysis for HPV‐16 incident infections and 12‐month PI also showed a somewhat lower risk in seropositive than seronegative women although the difference was not statistically significant (HR = 0.81 [0.56‐1.16; *P* = 0.26] and 0.53 [0.24‐1.16; *P* = 0.11], respectively). With regard to HPV‐18, we found the risk of newly detected infections and cervical abnormalities was lower in seropositive vs seronegative women, but not statistically significant (HR = 0.95 [0.59‐1.51; *P* = 0.82] for incident infections, 0.43 [0.16‐1.13; *P* = 0.09] for 6‐month PI, 0.31 [0.07‐1.36; *P* = 0.12] for 12‐month PI, and 0.61 [0.23‐1.61; *P* = 0.32] for ASC‐US+; Table 3). Other determinants (Tables 2 and 3, and Supplementary Tables) associated with a higher risk of new infections were ≥2 sexual partners during the past year (for incident HPV‐16 and HPV‐18 infections, and 6‐month and 12‐month HPV‐16 and HPV‐18 PI), being single (for incident HPV‐18 infections), a history of HPV infection/treatment, or having a nonintact cervix (for incident HPV‐16 and HPV‐18 infections). Women older than 35 years at enrollment had a lower risk of incident HPV‐16 infections as well as HPV‐18 incident infections, 6‐month PI, and ASC‐US+. The risk of infections varied significantly among geographical regions. The risk factors associated with ASC‐US+ were a history of HPV infection/treatment, a nonintact cervix (for HPV‐18), and a previous type‐specific HPV infection (for HPV‐16 and HPV‐18).

**Table 2:** Multivariable Cox model for HPV‐16 newly detected infections and cervical abnormalities including serostatus at baseline

Risk factor	Category	Enrollment serostatus (binary)															
		Incident infection				6‐mo PI				12‐mo PI				ASC‐US+			
		N	n	Hazard ratio (95% CI)	*P*‐value	N	n	Hazard ratio (95% CI)	*P*‐value	N	n	Hazard ratio (95% CI)	*P*‐value	N	n	Hazard ratio (95% CI)	*P*‐value
HPV‐16 serostatus	Negative	1814	114	1	‐	1779	67	1	*‐*	1755	38	1	*‐*	1787	43	1	‐
	Positive	790	45	0.81 (0.56‐1.16)	0.2559	767	17	**0.56 (0.32**‐**0.99)**	**0.0446**	753	8	0.53 (0.24‐1.16)	0.1123	**774**	**9**	**0.28 (0.12‐0.67)**	**0.0043**
Age at inclusion	26‐35	1157	94	1	‐	1134	45	1	*‐*	1109	21	1	*‐*	1139	33	1	‐
	≥36	**1157**	**65**	**0.58 (0.42**‐**0.82)**	**0.0016**	1412	39	0.78 (0.50‐1.24)	0.2946	1399	25	0.99 (0.54‐1.82)	0.9817	1423	19	0.57 (0.31‐1.03)	0.0640
Region	Europe	505	22	1	‐	495	6	1	‐	491	5	1	‐	500	8	1	‐
	Asia Pacific	779	38	1.19 (0.68‐2.08)	0.5445	**772**	**23**	**2.57 (1.01**‐**6.52)**	**0.0476**	765	15	1.75 (0.61‐5.05)	0.3004	772	10	0.77 (0.28‐2.10)	0.6065
	Latin America	679	43	1.56 (0.90‐2.71)	0.1142	**663**	**29**	**3.86 (1.54**‐**9.70)**	**0.0040**	658	16	2.26 (0.79‐6.48)	0.1308	666	18	1.66 (0.65‐4.21)	0.2862
	North America	**641**	**56**	**2.38 (1.42**‐**3.97)**	**0.0009**	**616**	**26**	**4.28 (1.74**‐**10.54)**	**0.0015**	594	10	1.89 (0.63‐5.66)	0.2575	623	16	1.32 (0.50‐3.46)	0.5716
Age at first sexual intercourse grouped	≥18	1654	98	1	‐	1621	57	1	‐	1600	33	1	‐	1630	32	1	‐
	15‐17	817	50	0.85 (0.58‐1.23)	0.3775	799	57	0.62 (0.36‐1.09)	0.0967	784	9	0.56 (0.26‐1.22)	0.1473	804	14	1.05 (0.53‐2.08)	0.8832
	<15	127	11	1.08 (0.56‐2.07)	0.8271	120	8	1.48 (0.67‐3.27)	0.3329	118	4	1.30 (0.43‐3.95)	0.6433	121	6	2.20 (0.79‐6.16)	0.1322
Marital status at baseline	Living or lived with partner	2227	129	1	‐	2177	69	1	‐	2151	41	1	‐	2190	44	1	‐
	Single	377	30	0.79 (0.49‐1.29)	0.3493	369	15	0.89 (0.46‐1.75)	0.7434	357	5	0.70 (0.24‐2.04)	0.5116	371	8	0.67 (0.27‐1.67)	0.3878
Smoking status at baseline	No	2264	131	1	‐	2221	72	1	*‐*	2192	40	1	*‐*	2229	40	1	‐
	Yes	340	28	1.35 (0.88‐2.06)	0.1701	325	12	1.15 (0.61‐2.17)	0.6678	316	6	1.10 (0.45‐2.70)	0.8278	332	12	1.78 (0.89‐3.59)	0.1049
Number of sexual partners during the last year	0	283	16	1	‐	277	8	1	‐	272	2	1	‐	279	5	1	‐
	1	2096	112	0.88 (0.52‐1.52)	0.6563	2052	59	1.02 (0.48‐2.18)	0.9634	2026	35	2.26 (0.54‐9.57)	0.2663	2064	36	0.89 (0.33‐2.35)	0.8105
	≥2	**225**	**31**	**2.36 (1.26**‐**4.44)**	**0.0074**	**217**	**17**	**3.53 (1.47**‐**8.48)**	**0.0048**	**210**	**9**	**8.20 (1.70**‐**39.49)**	**0.0087**	218	11	1.72 (0.56‐5.28)	0.3399
Pregnancy	No	401	35	1	‐	393	19	1	‐	384	7	1	‐	394	11	1	‐
	Yes	2203	124	0.74 (0.47‐1.14)	0.1732	2153	65	0.57 (0.31‐1.05)	0.0696	2124	39	0.82 (0.33‐2.06)	0.6793	2167	41	0.91 (0.40‐2.06)	0.8211
*Chlamydia* infection at baseline	No	2458	150	1	‐	2407	81	1	‐	2371	44	1	‐	2420	48	1	‐
	Yes	122	7	0.59 (0.27‐1.29)	0.1848	115	2	0.36 (0.08‐1.51)	0.1618	113	2	0.95 (0.21‐4.32)	0.9486	117	3	2.57 (0.71‐9.27)	0.1483
History of HPV infection/treatment or not intact cervix	No	2285	128	1	‐	2234	72	1	‐	2202	39	1	‐	2246	3	1	‐
	Yes	**319**	**31**	**1.56 (1.03**‐**2.35)**	**0.0348**	312	12	1.17 (0.62‐2.18)	0.6316	306	7	1.31 (0.57‐3.02)	0.5240	315	8	1.03 (0.45‐2.36)	0.9416
Previous HPV‐16 infection	No			‐	‐			‐	‐			‐	‐	2561	24	1	‐
	Yes			‐	‐			‐	‐			‐	‐	**128**	**28**	**122.89 (67.91**‐**222.37)**	**<.0001**

HPV = human papillomavirus; PI = persistent infection; CI = confidence interval; ACS‐US+ = atypical squamous cell of undetermined significance or greater; N = total number of subjects; n = number of cases reported. Bold: *P*‐values <0.05

**Table 3:** Multivariable Cox model for HPV‐18 newly detected infections and cervical abnormalities including serostatus at baseline

Risk factor	Category	Enrollment serostatus (binary)															
		Incident infection				6‐mo PI				12‐mo PI				ASC‐US+			
		N	n	Hazard ratio (95% CI)	*P*‐value	N	n	Hazard ratio (95% CI)	*P*‐value	N	n	Hazard ratio (95% CI)	*P*‐value	N	n	Hazard ratio (95% CI)	*P*‐value
HPV‐18 serostatus	Negative	1907	97	1	‐	1874	31	1	*‐*	1840	18	1	‐	1882	26	1	‐
	Positive	713	25	0.95 (0.59‐1.51)	0.8165	689	5	0.43 (0.16‐1.13)	0.0883	681	2	0.31 (0.07‐1.36)	0.1198	696	6	0.61 (0.23‐1.61)	0.3176
Age at inclusion	26‐35	1156	70	1	‐	1133	28	1	*‐*	1108	15	1	‐	1137	25	1	‐
	≥36	**1464**	**27**	**0.35 (0.22**‐**0.56)**	**<.0001**	**1430**	**8**	**0.29 (0.13**‐**0.65)**	**0.0029**	1413	5	0.39 (0.13‐1.15)	0.0863	**1441**	**7**	**0.32 (0.13**‐**0.77)**	**0.0115**
Region	Europe	518	19	1	‐	508	6	1	‐	502	7	1	‐	513	5	1	‐
	Asia Pacific	775	28	1.22 (0.65‐2.32)	0.5361	768	9	1.32 (0.41‐4.24)	0.6364	761	6	1.70 (0.38‐7.60)	0.4907	768	7	1.16 (0.31‐4.36)	0.8207
	Latin America	688	23	1.14 (0.58‐2.21)	0.7049	672	8	1.35 (0.41‐4.45)	0.6178	666	3	0.98 (0.18‐5.43)	0.9834	675	11	3.04 (0.82‐11.25)	0.0964
	North America	639	27	1.12 (0.60‐2.09)	0.7281	615	13	2.06 (0.71‐5.98)	0.1837	592	7	1.99 (0.49‐8.13)	0.3378	622	9	2.70 (0.73‐9.97)	0.1371
Age at first sexual intercourse grouped	≥18	1666	67	1	‐	1633	23	1	‐	1610	14	1	‐	1642	23	1	‐
	15‐17	821	31	0.96 (0.59‐1.55)	0.8591	804	12	0.81 (0.37‐1.78)	0.6062	787	6	0.71 (0.24‐2.09)	0.5349	809	6	0.41 (0.15‐1.12)	0.0818
	<15	126	9	1.57 (0.74‐3.34)	0.2430	119	1	0.37 (0.05‐2.91)	0.3439	117	0	Not estimated	‐	120	3	1.82 (0.46‐7.24)	0.3945
Marital status at baseline	Living or lived with partner	2234	67	1	‐	2185	24	1	‐	2156	12	1	‐	2198	22	1	‐
	Single	**385**	**30**	**2.13 (1.22**‐**3.71)**	**0.0076**	377	12	1.96 (0.79‐4.86)	0.1452	364	8	2.79 (0.82‐9.48)	0.1010	379	10	1.66 (0.58‐4.71)	0.3426
Smoking status at baseline	No	2270	82	1	‐	2228	29	1	*‐*	2196	16	1	‐	2236	28	1	‐
	Yes	349	15	0.88 (0.49‐1.58)	0.6754	334	7	1.33 (0.56‐3.14)	0.5190	324	4	1.43 (0.45‐4.51)	0.5416	341	4	0.78 (0.26‐2.35)	0.6547
Number of sexual partners during the last year	0	291	7	1	‐	285	2	1	‐	280	2	1	‐	287	3	1	‐
	1	2097	68	1.46 (0.65‐3.26)	0.3565	2054	25	1.85 (0.42‐8.16)	0.4151	2026	13	1.09 (0.22‐5.27)	0.9169	2066	23	1.23 (0.34‐4.36)	0.7530
	≥2	**231**	**22**	**3.28 (1.36**‐**7.88)**	**0.0080**	223	9	4.69 (0.97‐22.56)	0.0540	214	5	2.92 (0.53‐16.23)	0.2197	224	6	1.57 (0.35‐7.15)	0.5589
Pregnancy	No	404	21	1	‐	396	9	1	‐	386	6	1	‐	397	8	1	‐
	Yes	2215	76	1.17 (0.66‐2.06)	0.5931	2166	27	1.14 (0.46‐2.83)	0.7715	2134	14	1.03 (0.32‐3.35)	0.9621	2180	24	0.96 (0.32‐2.87)	0.9462
*Chlamydia* infection at baseline	No	2477	89	1	‐	2427	33	1	‐	2388	18	1	‐	2440	30	1	‐
	Yes	118	6	0.82 (0.34‐1.96)	0.6579	111	2	0.80 (0.18‐3.52)	0.7643	109	1	0.73 (0.09‐5.82)	0.7666	113	1	0.27 (0.03‐2.53)	0.2534
History of HPV infection/treatment or not intact cervix	No	2285	75	1	‐	2236	27	1	‐	2203	1	1	‐	2248	22	1	‐
	Yes	**335**	**22**	**1.72 (1.03**‐**2.86)**	**0.0373**	327	9	1.79 (0.79‐4.09)	0.1658	318	5	1.64 (0.52‐5.20)	0.3980	**330**	**10**	**2.57 (1.10**‐**6.01)**	**0.0288**
Previous cervical HPV‐18 infection	No			‐	‐			‐	‐			‐	‐	2578	20	1	‐
	Yes			‐	‐			‐	‐			‐	‐	**68**	**12**	**122.93 (54.69**‐**276.33)**	**<.0001**

HPV = human papillomavirus; PI = persistent infection; CI = confidence interval; ACS‐US+ = atypical squamous cell of undetermined significance or greater; N = total number of subjects; n = number of cases reported. Bold: *P*‐values <0.05

**Figure 2:**
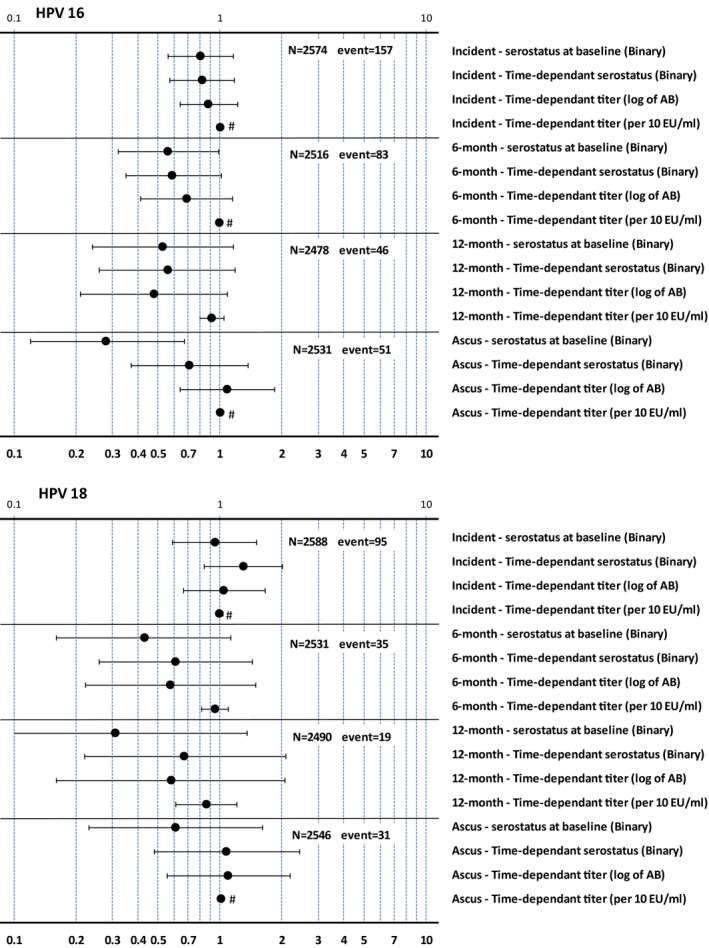
Risk ratio of incident, 6‐mo persistent, and 12‐mo persistent infection and atypical squamous cell of undetermined significance or greater in HPV‐16/HPV‐18 type‐specific seropositive vs seronegative women. Error bars represent 95% confidence intervals; ^#^95% confidence intervals are narrow and not visible; HPV, human papillomavirus; PI, persistent infection; bin, binary; ab, antibody; ACS‐US+, atypical squamous cell of undetermined significance or greater

The other multivariable Cox proportional hazard models (including the serostatus as a time‐dependent variable, antibody level as a time‐dependent variable, and log‐transformed level as a time‐dependent variable) showed similar results (Figure 2 and Supplementary Tables).

The analyses stratified by baseline serostatus showed that these risk factors (number of sexual partners in the last 12 months, living single and smoking) were more marked in seronegative than in seropositive women (Table 4).

**Table 4:** Multivariable Cox model for HPV‐16 and HPV‐18 6‐mo persistent infection and incident infection according to different serostatus at baseline

Risk factor	HPV‐16 6‐mo PI				HPV‐18 6‐mo PI				HPV‐16 incident infection				HPV‐18 incident infection			
	Seronegative		Seropositive		Seronegative		Seropositive		Seronegative		Seropositive		Seronegative		Seropositive	
	N = 1767 event=67		N = 749 event=16		N = 1852 event=30		N = 679 event=5		N = 1802 event=114		N = 772 event=43		N = 1885 event =70		N = 703 event=25	
	Hazard ratio (95% CI)	*P*‐value	Hazard ratio (95% CI)	*P*‐value	Hazard ratio (95% CI)	*P*‐value	Hazard ratio (95% CI)	*P*‐value	Hazard ratio (95% CI)	*P*‐value	Hazard ratio (95% CI)	*P*‐value	Hazard ratio (95% CI)	*P*‐value	Hazard ratio (95% CI)	*P*‐value
Age at inclusion																
26‐35	1	‐	1	‐	1	‐	1	‐	1	‐	1	‐	1	‐	1	‐
≥36	0.73 (0.44‐1.22)	0.2306	1.27 (0.43‐3.74)	0.6625	**0.33 (0.14**‐**0.77)**	**0.0103**	Not estimated	‐	**0.62 (0.42‐0.92)**	**0.0177**	**0.51 (0.27‐0.97)**	**0.0409**	**0.33 (0.19‐0.57)**	**<0.001**	0.41 (0.17‐1.01)	0.0527
Region																
Europe	1	‐	1	‐	1	‐	1	‐	1	‐	1	‐	1	‐	1	‐
Asia Pacific	2.70 (0.88‐8.29)	0.0825	2.79 (0.50‐15.71)	0.2447	1.16 (0.35‐3.81)	0.8069	Not estimated	‐	1.13 (0.59‐2.13)	0.7172	1.25 (0.37‐4.21)	0.7169	1.06 (0.48‐2.31)	0.8893	1.85 (0.57‐5.95)	0.3033
Latin America	**5.00 (1.66**‐**15.02)**	**0.0042**	1.33 (0.18‐10.04)	0.7804	0.83 (0.23‐2.95)	0.7750	Not estimated	‐	1.46 (0.77‐2.76)	0.2433	2.03 (0.65‐6.36)	0.2225	1.07 (0.49‐2.33)	0.8717	1.41 (0.38‐5.25)	0.6131
North America	**5.27 (1.77**‐**15.73)**	**0.0029**	2.80 (0.58‐13.52)	0.2002	1.74 (0.57‐5.28)	0.3319	Not estimated	‐	**2.27 (1.24‐4.17)**	**0.0081**	**2.76 (1.03‐7.43)**	**0.0445**	1.44 (0.69‐3.00)	0.3268	0.59 (0.18‐2.01)	0.4022
Age at first sexual intercourse grouped																
≥18	1	‐	1	‐	1	‐	1	‐	1	‐	1	‐	1	‐	1	‐
15‐17	0.58 (0.30‐1.09)	0.0915	0.67 (0.20‐2.29)	0.5274	0.67 (0.28‐1.60)	0.3669	5.52 (0.37‐81.42)	0.2133	0.81 (0.52‐1.26)	0.3457	0.88 (0.43‐1.81)	0.7343	0.97 (0.56‐1.68)	0.9072	0.79 (0.29‐2.15)	0.6378
<15	0.86 (0.29‐2.55)	0.7829	**4.04 (1.06**‐**15.47)**	**0.0413**	Not estimated	‐	29.71 (0.70‐1263.78)	0.0763	0.58 (0.20‐1.64)	0.3042	1.77 (0.69‐4.54)	0.2372	0.78 (0.23‐2.63)	0.6868	**3.58 (1.19‐10.83)**	**0.0237**
Marital status at baseline																
Living or lived with partner	1	‐	1	‐	1	‐	1	‐	1	‐	1	‐	1	‐	1	‐
Single	0.97 (0.45‐2.11)	0.9381	0.87 (0.22‐3.46)	0.8395	1.93 (0.70‐5.36)	0.2039	2.73 (0.20‐36.42)	0.4484	0.75 (0.42‐1.37)	0.3537	1.02 (0.45‐2.30)	0.9587	1.93 (1.00‐3.73)	0.0514	2.64 (0.93‐7.44)	0.0672
Smoking status at baseline																
No	1	‐	1	‐	1	‐	1	‐	1	‐	1	‐	1	‐	1	‐
Yes	1.36 (0.68‐2.74)	0.3825	0.68 (0.15‐3.17)	0.6283	1.40 (0.55‐3.58)	0.4801	0.84 (0.07‐9.42)	0.8874	**1.75 (1.07‐2.86)**	**0.0247**	0.72 (0.29‐1.74)	0.4633	1.03 (0.59‐3.92)	0.9385	0.57 (0.16‐1.98)	0.3747
Number of sexual partners during the last year																
0	1	‐	1	‐	1	‐	1	‐	1	‐	1	‐	1	‐	1	‐
1	1.08 (0.45‐2.59)	0.8652	0.72 (0.15‐3.52)	0.6853	1.58 (0.35‐7.15)	0.5522	Not estimated	‐	0.84 (0.45‐1.57)	0.5839	0.99 (0.34‐2.92)	0.9919	1.52 (0.59‐3.92)	0.3916	1.33 (0.28‐6.22)	0.7199
≥2	**4.49 (1.62**‐**12.49)**	**0.0039**	2.04 (0.33‐12.59)	0.4428	4.51 (0.90‐22.75)	0.0679	Not estimated	‐	**2.60 (1.23‐5.50)**	**0.0127**	1.98 (0.59‐6.69)	0.2704	**3.36 (1.18‐9.63)**	**0.0238**	3.36 (0.67‐16.91)	0.1419
Pregnancy																
No	1	‐	1	‐	1	‐	1	‐	1	‐	1	‐	1	‐	1	‐
Yes	0.59 (0.30‐1.19)	0.1396	0.57 (0.15‐2.22)	0.4207	1.74 (0.60‐5.01)	0.3044	0.10 (0.01‐1.72)	0.1119	**0.60 (0.36‐1.01)**	**0.0127**	1.44 (0.57‐3.63)	0.4343	1.40 (0.70‐2.79)	0.3415	0.70 (0.25‐1.97)	0.4954
*Chlamydia* infection at baseline																
No	1	‐	1	‐	1	‐	1	‐	1	‐	1	‐	1	‐	1	‐
Yes	Not estimated	‐	0.85 (0.18‐4.06)	0.8404	0.59 (0.08‐4.55)	0.6120	1.97 (0.15‐26.31)	0.6071	0.17 (0.02‐1.22)	0.0777	1.09 (0.44‐2.72)	0.8547	0.73 (0.22‐2.45)	0.6094	1.03 (0.26‐4.12)	0.9658
History of HPV infection/treatment or not intact cervix																
No	1	‐	1	‐	1	‐	1	‐	1	‐	1	‐	1	‐	1	‐
Yes	0.87 (0.39‐1.94)	0.7329	1.74 (0.58‐5.22)	0.3202	2.10 (0.86‐5.13)	0.1024	0.76 (0.07‐8.56)	0.8229	1.36 (0.80‐2.32)	0.2582	1.89 (0.96‐3.71)	0.0648	1.82 (0.99‐3.35)	0.0530	1.19 (0.43‐3.30)	0.7309

HPV = human papillomavirus N = number of subjects used in the model; event = number of HPV‐type‐specific 6‐mo persistent cervical infection; PI = persistent infection; CI = confidence interval. Bold: *P*‐values <0.05

## 4 DISCUSSION

In this study, HPV‐16‐seropositive women of 25 years and older had a moderate decrease in risk of developing a new type‐specific HPV, PI, and ASC‐US+ compared to seronegative women. This result agrees with the hypothesis that naturally acquired HPV antibodies probably provide only partial protection against subsequent infection with the same HPV type. However, HPV‐18‐seropositive women had deficient levels of protection. Any naturally acquired protection afforded by either antibody is unlikely to be better than the benefits acquired by vaccination. Another study has found that women aged between 30 and 50 who were seropositive for high risk (HR) HPV at baseline had a higher incidence of new type‐specific HPV infection than women who were seronegative.^26^

The association between seropositivity and the reduced risk of new infection was less in our study of 26+‐year‐old women than demonstrated in our study of younger women aged 15‐25 years in PATRICIA and in the Costa Rica Vaccine Trial.^12^, ^15^ This low protective effect or even absence of protective effect in >25‐year‐old women could suggest waning of the natural immunity but it could also reflect reactivation of prior infection.^26^

In the present study, we were not able to determine an accurate antibody threshold value for a defined reduction rate in infection. In the PATRICIA trial, HPV‐16 antibody levels comprised between 200 and 500 EU/mL were associated with a 90% reduction of incident infection, of 6‐month PI and of ASC‐US+.^12^ For HPV‐18, seropositivity was associated with a lower risk of ASC‐US+ and CIN1+ but no association was found between naturally acquired antibodies and new infection.^12^

The current study also attempted to consider the change in serostatus during the follow‐up period. Including the serostatus as a time‐dependent variable and as a continuous variable in the Cox models is original. In a recent meta‐analysis, assessing the naturally acquired immunity against HPV infection, none of the 14 included studies considered the possible change of serostatus during the follow‐up period.^27^ Overall, our various models gave consistent results. However, the interpretation of the time‐dependent serostatus models can be challenging because of the interaction between the change in antibody titers and the incidence of new HPV infections. Because the serology was collected every 12 months and the cervical sample every six months, new, but undetected, infection could have boosted the antibody titer.

In another analysis of the control cohort of the VIVIANE trial, the risk of detecting CIN after natural HPV infection in women aged >25 years was similar to that observed in women aged 15‐25 years from the PATRICIA trial.^24^ This observation suggests that there are little to no age‐related differences in the detection of natural HPV infection and their associated CIN lesions.

Our analysis of determinants when considered separately for the baseline seronegative and seropositive subjects partially supports the hypothesis suggested by other studies that most of the newly detected HPV infections in seropositive women would be a reactivation of prior HPV infections.^19^, ^20^

The strengths of this study included the large cohort size of approximately 2700 women, and the relatively extended follow‐up period of seven years, which allowed for a thorough evaluation of an unvaccinated cohort. This study also had several limitations. A cervical sample test was performed only every six months, which could have meant that some incident HPV infections were not detected. In addition, it was not possible to determine whether an infection was quiescent, persistent at undetectable levels or was a new infection. Evidence exists that type‐specific HPV infection can present after a period of nondetection.^28^ Based on this assumption, some infections considered as new could indeed be a PI. This scenario could also bias the assessment of the relationship between natural antibodies and risk of new infection. Furthermore, the number of CIN1+ and CIN2+ cases was too low to allow for inferential analyses. Since we were unable to define which HPV type caused the abnormal cytology, ASC‐US+ lesions could ensue from non‐HPV‐16/18 types.

Further research is needed to better understand the natural history of HPV infection and the link between seropositivity and subsequent protection in women of different age groups.

In conclusion, multivariable Cox analyses showed evidence of lower risk of newly detected incident and persistent HPV infections and ASC‐US+ in women with naturally acquired antibodies against HPV‐16. The results for HPV‐18 are not conclusive since only a limited and nonsignificant decrease in risk was observed. These findings are consistent with a partial protective role of naturally acquired HPV antibodies against future infection with the corresponding HPV type. However, no threshold of antibody levels necessary for protection could be defined.

## **CONFLICT** **OF INTEREST**

D Rosillon and F Struyf are employed by the GSK group of companies and received GSK shares. L Baril was employed by the GSK group of companies at the time of the study and received GSK shares. G Dubin is currently a full‐time employee of Takeda Pharmaceuticals, Deerfield, Illinois, and receives salary and stock shares. MR Del Rosario‐Raymundo reports payment of honorarium as principal investigator and support for travel to meetings for the study from the GSK group of companies during the conduct of the study; payment for lectures including service on speakers' bureaus from the GSK group of companies. M Martens reports grants from the GSK group of companies, during the conduct of the study. C Bouchard reports grants from the GSK group of companies, during the conduct of the study. She reports grants and honorarium from Merck. KL Fong reports grant from the GSK group of companies via her institution for the conduct of the study. MC Bozonnat is a consultant outsourced from 4Clinics to the GSK group of companies. A Chatterjee received grant funding for clinical trials, and served on the speakers' bureau and advisory boards for the GSK group of companies and Merck. SM Garland has received advisory board fees and grants from CSL and the GSK group of companies, and lectures fees from Merck, the GSK group of companies, and Sanofi Pasteur. In addition, she received funding through her institution to conduct HPV vaccines studies for MSD and the GSK group of companies. She is a member of the Merck Global Advisory Board as well as the Merck Scientific Advisory Committee for HPV. E Lazcano‐Ponce received fees to conduct HPV vaccines studies from the GSK group of companies and Merck. SA McNeil has received research grants from the GSK group of companies and Sanofi Pasteur and speaker honoraria from Merck. B Romanowski received research grants, travel support, and speaker honoraria from the GSK group of companies. SR Skinner received funds through her institution from the GSK group of companies to cover expenses involved in the collection of data for this study. The GSK group of companies provided funds to reimburse expenses incurred with travel to conference to present data from other studies and paid honoraria to her institution for work conducted in the context of Advisory Board and educational meetings. CM Wheeler's institution received a contract from the GSK group of companies to act as a clinical trial site for this study, and reimbursements for travel related to publication activities and for HPV vaccine studies. Her institution also received funding from Merck to conduct HPV vaccine trials, and from Roche Molecular Systems equipment and reagents for HPV genotyping studies, outside the submitted work. X Castellsagué received research funding through his institution (ICO) from Merck & Co, SPMSD, the GSK group of companies, and Genticel. He also received honoraria for conferences from Vianex and SPMSD. G Minkina, as an investigator at a study clinical site, received fees from the GSK group of companies through her institution. She also received funding from Merck Sharp & Dohme to participate as principal investigator in efficacy trials. She received travel support to attend scientific meetings, honoraria for speaking engagements and participation in advisory board meetings, and consulting fees from the GSK group of companies and Merck Sharp & Dohme. T Stoney received honoraria from the GSK group of companies for study committee membership (Asia Pacific study follow‐up committee for Zoster studies), for conference attendance, and travel support. Her institution also received additional funding from a bioCSL grant for a project in which she is an investigator, funded by National Health and Medical Research Council. She also received travel support for participation in study investigator meetings from Novartis Vaccine and Diagnostics, Sanofi Pasteur, Alios BioPharma, and Pfizer. SC Quek received honoraria and travel expenses from the GSK group of companies for speaking at various symposia. A Savicheva received grants and fees from the GSK group of companies to participate in an epidemiological study (HERACLES). J Salmeron received grants from the GSK group of companies, Qiagen, and Merck Inc. D Money has received grants from Merck, the GSK group of companies, Novartis, and Sanofi for studies conduct. CS Vallejos, TYK Lim, B ter Harmsel, M Cruickshank, A Fiander, and A Ilancheran have nothing to disclose.
