# Supplementary material for: Risk of newly detected infections and cervical abnormalities in adult women seropositive or seronegative for naturally acquired HPV‐16/18 antibodies
Source: Cancer Med. 2019 Jul 5;8(10):4938–53. doi: 10.1002/cam4.1879 (PMC6712465; doi:10.1002/cam4.1879)
Supplement: Supplementary file 1 [file CAM4-8-4938-s001.docx]

**Supplementary tables**

**Table S1.** Multivariable Cox model for risk of newly detected human papillomavirus type 16 (HPV-16) infections or cervical abnormalities associated with HPV-16 including serostatus as time-dependent variable

|  |  | **Enrolment serostatus (time-dependent)** | | | | | | | |
| --- | --- | --- | --- | --- | --- | --- | --- | --- | --- |
|  |  | **Incident infection** | | **6-month PI** | | **12-month PI** | | **ASC-US+** | |
| Risk factor | Category | **Hazard ratio  (95% CI)** | **p-value** | **Hazard ratio  (95% CI)** | **p-value** | **Hazard ratio  (95% CI)** | **p-value** | **Hazard ratio  (95% CI)** | **p-value** |
| HPV-16 serostatus | Negative | 1 | - | 1 | - | 1 | - | 1 | - |
|  | Positive | 0.82 (0.57–1.17) | 0.2718 | 0.59 (0.35–1.02) | 0.0598 | 0.56 (0.26–1.19) | 0.1331 | 0.71 (0.37–1.37) | 0.3033 |
| Age at inclusion | 26–35 | 1 | - | 1 | - | 1 | - | 1 | - |
|  | ≥36 | **0.58 (0.42–0.82)** | **0.0016** | 0.78 (0.50–1.24) | 0.2959 | 0.99 (0.54–1.82) | 0.9847 | 0.57 (0.31–1.04) | 0.0677 |
| Region | Europe | 1 | - | 1 | - | 1 | - | 1 | - |
|  | Asia Pacific | 1.18 (0.68–2.07) | 0.5536 | **2.54 (1.00–6.47)** | **0.0499** | 1.75 (0.61–5.07) | 0.2990 | 0.77 (0.28–2.10) | 0.6066 |
|  | Latin America | 1.56 (0.90–2.71) | 0.1168 | **3.82 (1.52–9.61)** | **0.0043** | 2.27 (0.79–6.51) | 0.1288 | 1.68 (0.66–4.24) | 0.2746 |
|  | North America | **2.38 (1.42–3.98)** | **0.0009** | **4.27 (1.74–10.50)** | **0.0016** | 1.90 (0.63–5.70) | 0.2526 | 1.10 (0.42–2.88) | 0.8426 |
| Age at first sexual intercourse grouped | ≥18 | 1 | - | 1 | - | 1 | - | 1 | - |
|  | 15-17 | 0.85 (0.58–1.23) | 0.3750 | 0.62 (0.36–1.09) | 0.0946 | 0.56 (0.26–1.21) | 0.1414 | 1.00 (0.51–1.97) | 0.9980 |
|  | <15 | 1.07 (0.55–2.05) | 0.8490 | 1.45 (0.66–3.18) | 0.3608 | 1.26 (0.4– 3.81) | 0.6865 | 1.65 (0.60–4.55) | 0.3340 |
| Marital status at baseline | Living or lived with partner | 1 | - | 1 | - | 1 | - | 1 | - |
|  | Single | 0.79 (0.49–1.28) | 0.3383 | 0.88 (0.45–1.73) | 0.7100 | 0.68 (0.23–2.00) | 0.4857 | 0.68 (0.27–1.71) | 0.4093 |
| Smoking status at baseline | No | 1 | - | 1 | - | 1 | - | 1 | - |
|  | Yes | 1.35 (0.88–2.06) | 0.1723 | 1.14 (0.61–2.15) | 0.6818 | 1.10 (0.45–2.68) | 0.8404 | 1.70 (0.83–3.47) | 0.1460 |
| Number of sexual partners during the last year | 0 | 1 | - | 1 | - | 1 | - | 1 | - |
|  | 1 | 0.88 (0.51–1.52) | 0.6529 | 1.02 (0.48–2.17) | 0.9671 | 2.27 (0.54–9.59) | 0.2639 | 0.86 (0.33–2.26) | 0.7570 |
|  | ≥2 | **2.38 (1.27–4.48)** | **0.0068** | **3.61 (1.50–8.68)** | **0.0041** | **8.55 (1.77–41.21)** | **0.0075** | 1.56 (0.50–4.86) | 0.4439 |
| Pregnancy | No | 1 | - | 1 | - | 1 | - | 1 | - |
|  | Yes | 0.74 (0.47–1.14) | 0.1736 | 0.57 (0.31–1.04) | 0.0671 | 0.81 (0.33–2.04) | 0.6616 | 0.88 (0.40–1.98) | 0.7659 |
| Chlamydia infection at baseline | No | 1 | - | 1 | - | 1 | - | 1 | - |
|  | Yes | 0.59 (0.27–1.29) | 0.1850 | 0.36 (0.08–1.50) | 0.1595 | 0.95 (0.21–4.33) | 0.9503 | 2.33 (0.65–8.43) | 0.1961 |
| History of HPV infection/treatment or not intact cervix | No | 1 | - | 1 | - | 1 | - | 1 | - |
|  | Yes | **1.55 (1.03–2.34)** | **0.0357** | 1.16 (0.62–2.17) | 0.6425 | 1.32 (0.57–3.02) | 0.5182 | 0.98 (0.43–2.25) | 0.9644 |
| Previous cervical HPV-16 infection | No | - | - | - | - | - | - | 1 | - |
|  | Yes | - | - | - | - | - | - | **119.43 (66.28–215.22)** | **<.0001** |

HPV = human papillomavirus N = number of subjects used in the model; event = number of HPV-type specific 6-month persistent cervical infection; PI = persistent infection; CI = confidence interval. **Bold:** p-values < 0.05

**Table S2.** Multivariable Cox model for risk of newly detected human papillomavirus type 18 (HPV-18) infections or cervical abnormalities associated with HPV-18 including serostatus as time-dependent variable

|  |  | **Enrolment serostatus (time-dependent)** | | | | | | | |
| --- | --- | --- | --- | --- | --- | --- | --- | --- | --- |
|  |  | **Incident infection** | | **6-month PI** | | **12-month PI** | | **ASC-US+** | |
| Risk factor | Category | **Hazard ratio  (95% CI)** | **p-value** | **Hazard ratio  (95% CI)** | **p-value** | **Hazard ratio  (95% CI)** | **p-value** | **Hazard ratio  (95% CI)** | **p-value** |
| HPV-18 serostatus | Negative | 1 | - | 1 | - | 1 | - | 1 | - |
|  | Positive | 1.30 (0.84–2.01) | 0.2365 | 0.61 (0.26–1.43) | 0.2560 | 0.67 (0.22–2.09) | 0.4916 | 1.08 (0.48–2.44) | 0.8528 |
| Age at inclusion | 26–35 | 1 | - | 1 | - | 1 | - | 1 | - |
|  | ≥36 | **0.34 (0.22–0.55)** | **<.0001** | **0.28 (0.12–0.64)** | **0.0026** | 0.38 (0.13–1.11) | 0.0771 | **0.31 (0.13–0.75)** | **0.0094** |
| Region | Europe | 1 | - | 1 | - | 1 | - | 1 | - |
|  | Asia Pacific | 1.24 (0.65–2.34) | 0.5150 | 1.27 (0.40– 4.03) | 0.6884 | 1.59 (0.36–7.02) | 0.5389 | 1.07 (0.29–3.91) | 0.9243 |
|  | Latin America | 1.16 (0.60–2.26) | 0.6611 | 1.34 (0.41–4.39) | 0.6301 | 0.97 (0.18–5.31) | 0.9712 | 2.83 (0.79–10.18) | 0.1104 |
|  | North America | 1.11 (0.59–2.08) | 0.7388 | 2.02 (0.70–5.87) | 0.1959 | 1.93 (0.47–7.87) | 0.3610 | 2.47 (0.68–9.02) | 0.1714 |
| Age at first sexual intercourse grouped | ≥18 | 1 | - | 1 | - | 1 | - | 1 | - |
|  | 15-17 | 0.93 (0.57–1.50) | 0.7617 | 0.80 (0.37–1.75) | 0.5804 | 0.68 (0.23–1.99) | 0.4758 | 0.38 (0.14–1.05) | 0.0633 |
|  | <15 | 1.52 (0.71–3.23) | 0.2773 | 0.34 (0.04–2.72) | 0.3115 | Not estimated | - | 1.65 (0.41–6.58) | 0.4811 |
| Marital Status at baseline | Living or lived with partner | 1 | - | 1 | - | 1 | - | 1 | - |
|  | Single | **2.13 (1.22–3.70)** | **0.0078** | 1.98 (0.80–4.89) | 0.1412 | 2.80 (0.83–9.49) | 0.0980 | 1.63 (0.57–4.69) | 0.3662 |
| Smoking status at baseline | No | 1 | - | 1 | - | 1 | - | 1 | - |
|  | Yes | 0.88 (0.49–1.58) | 0.6700 | 1.33 (0.56 –3.14) | 0.5177 | 1.45 (0.46–4.56) | 0.5244 | 0.82 (0.27–2.47) | 0.7261 |
| Number of sexual partners during the last year | 0 | 1 | - | 1 | - | 1 | - | 1 | - |
|  | 1 | 1.46 (0.65–3.26) | 0.3564 | 1.84 (0.42–8.06) | 0.4202 | 1.06 (0.22–5.06) | 0.9445 | 1.22 (0.34–4.31) | 0.7607 |
|  | ≥2 | **3.22 (1.34–7.74)** | **0.0089** | 4.58 (0.95–22.01) | 0.0575 | 2.77 (0.50 - 15.27) | 0.2431 | 1.41 (0.31 - 6.43) | 0.6542 |
| Pregnancy | No | 1 | - | 1 | - | 1 | - | 1 | - |
|  | Yes | 1.17 (0.66–2.06) | 0.5937 | 1.14 (0.46–2.82) | 0.7749 | 1.04 (0.32–3.37) | 0.9522 | 1.01 (0.34–3.05) | 0.9841 |
| Chlamydia infection at baseline | No | 1 | - | 1 | - | 1 | - | 1 | - |
|  | Yes | 0.80 (0.33–1.91) | 0.6099 | 0.76 (0.17–3.33) | 0.7132 | 0.66 (0.08–5.25) | 0.6951 | 0.23 (0.02–2.13) | 0.1939 |
| History of HPV infection/treatment or not intact cervix | No | 1 | - | 1 | - | 1 | - | 1 | - |
|  | Yes | **1.68 (1.01–2.80)** | **0.0468** | 1.73 (0.76–3.94) | 0.1949 | 1.52 (0.48–4.81) | 0.4791 | **2.52 (1.08–5.87)** | **0.0322** |
| Previous cervical HPV-18 infection | No | - | - | - | - | - | - | 1 | - |
|  | Yes | - | - | - | - | - | - | **123.17 (54.37–279.00)** | **<.0001** |

HPV = human papillomavirus N = number of subjects used in the model; event = number of HPV-type specific 6-month persistent cervical infection; PI = persistent infection; CI = confidence interval. **Bold:** p-values < 0.05

**Table S3.** Multivariable Cox model for risk of newly detected human papillomavirus type 16 (HPV-16) infections or cervical abnormalities associated with HPV-16 including log-transformed antibody level as time-dependent variable

|  |  | **Log of antibody level (time-dependent)** | | | | | | | |
| --- | --- | --- | --- | --- | --- | --- | --- | --- | --- |
|  |  | **Incident infection** | | **6-month PI** | | **12-month PI** | | **ASC-US+** | |
| Risk factor | Category | **Hazard ratio  (95% CI)** | **p-value** | **Hazard ratio  (95% CI)** | **p-value** | **Hazard ratio  (95% CI)** | **p-value** | **Hazard ratio  (95% CI)** | **p-value** |
| HPV-16 log of antibody level as time-dependent |  | 0.8840 (0.6420–1.2174) | 0.4503 | 0.6895 (0.4134–1.1497) | 0.1541 | 0.4813 (0.2132–1.0864) | 0.0783 | 1.0892 (0.6425–1.8464) | 0.7511 |
| Age at inclusion | 26–35 | 1 | - | 1 | - | 1 | - | 1 | - |
|  | ≥36 | **0.58 (0.42–0.81)** | **0.0015** | 0.78 (0.50–1.23) | 0.2844 | 1.00 (0.55–1.82) | 0.9913 | 0.57 (0.31–1.05) | 0.0728 |
| Region | Europe | 1 | - | 1 | - | 1 | - | 1 | - |
|  | Asia Pacific | 1.19 (0.68–2.09) | 0.5335 | **2.59 (1.02–6.59)** | **0.0453** | 1.73 (0.60–5.00) | 0.3103 | 0.79 (0.29–2.16) | 0.6460 |
|  | Latin America | 1.57 (0.90–2.73) | 0.1107 | **3.88 (1.54–9.74)** | **0.0039** | 2.23 (0.78–6.39) | 0.1369 | 1.72 (0.68–4.34) | 0.2520 |
|  | North America | **2.38 (1.42–3.98)** | **0.0009** | **4.29 (1.74–10.55)** | **0.0015** | 1.92 (0.64–5.77) | 0.2447 | 1.04 (0.40–2.72) | 0.9312 |
| Age at first sexual intercourse grouped | ≥18 | 1 | - | 1 | - | 1 | - | 1 | - |
|  | 15-17 | 0.84 (0.58–1.22) | 0.3687 | 0.62 (0.36–1.08) | 0.0926 | 0.57 (0.26–1.25) | 0.1600 | 0.96 (0.49–1.90) | 0.9095 |
|  | <15 | 1.06 (0.55–2.05) | 0.8548 | 1.45 (0.66–3.19) | 0.3576 | 1.26 (0.41–3.83) | 0.6829 | 1.56 (0.56–4.31) | 0.3936 |
| Marital Status at baseline | Living or lived with partner | 1 | - | 1 | - | 1 | - | 1 | - |
|  | Single | 0.78 (0.48–1.27) | 0.3234 | 0.86 (0.44–1.69) | 0.6673 | 0.68 (0.23–1.99) | 0.4786 | 0.69 (0.28–1.72) | 0.4268 |
| Smoking status at baseline | No | 1 | - | 1 | - | 1 | - | 1 | - |
|  | Yes | 1.34 (0.87–2.05) | 0.1800 | 1.13 (0.60–2.12) | 0.7144 | 1.07 (0.44–2.62) | 0.8802 | 1.65 (0.81–3.38) | 0.1695 |
| Number of sexual partners during the last year | 0 | 1 | - | 1 | - | 1 | - | 1 | - |
|  | 1 | 0.88 (0.51–1.52) | 0.6521 | 1.01 (0.47–2.16) | 0.9777 | 2.26 (0.54–9.55) | 0.2668 | 0.86 (0.33–2.27) | 0.7659 |
|  | ≥2 | **2.37 (1.26–4.44)** | **0.0073** | **3.55 (1.48–8.52)** | **0.0046** | **8.59 (1.78–41.45)** | **0.0074** | 1.46 (0.47–4.53) | 0.5167 |
| Pregnancy | No | 1 | - | 1 | - | 1 | - | 1 | - |
|  | Yes | 0.73 (0.47–1.14) | 0.1666 | 0.56 (0.31–1.02) | 0.0597 | 0.81 (0.32–2.03) | 0.6536 | 0.88 (0.39–1.98) | 0.7667 |
| Chlamydia infection at baseline | No | 1 | - | 1 | - | 1 | - | 1 | - |
|  | Yes | 0.59 (0.27–1.29) | 0.1829 | 0.36 (0.08–1.50) | 0.1598 | 1.02 (0.22–4.64) | 0.9836 | 2.15 (0.59–7.80) | 0.2453 |
| History of HPV infection/treatment or not intact cervix | No | 1 | - | 1 | - | 1 | - | 1 | - |
|  | Yes | **1.55 (1.03–2.34)** | **0.0358** | 1.16 (0.62–2.17) | 0.6394 | 1.34 (0.58–3.07) | 0.4916 | 0.97 (0.42–2.23) | 0.9390 |
| Previous cervical HPV-16 infection | No | - | - | - | - | - | - | 1 | - |
|  | Yes | - | - | - | - | - | - | **116.56 (64.73–209.91)** | **<.0001** |

HPV = human papillomavirus N = number of subjects used in the model; event = number of HPV-type specific 6-month persistent cervical infection; PI = persistent infection; CI = confidence interval. **Bold:** p-values < 0.05

**Table S4.** Multivariable Cox model for risk of newly detected human papillomavirus type 18 (HPV-18) infections or cervical abnormalities associated with HPV-18 including log-transformed antibody level as time-dependent variable

|  |  | **Log of antibody level (time-dependent)** | | | | | | | |
| --- | --- | --- | --- | --- | --- | --- | --- | --- | --- |
|  |  | **Incident infection** | | **6-month PI** | | **12-month PI** | | **ASC-US+** | |
| Risk factor | Category | **Hazard ratio  (95% CI)** | **p-value** | **Hazard ratio  (95% CI)** | **p-value** | **Hazard ratio  (95% CI)** | **p-value** | **Hazard ratio  (95% CI)** | **p-value** |
| HPV-18 log of antibody level as time-dependent |  | 1.0485 (0.6629–1.6584) | 0.8397 | 0.5740 (0.2217–1.4860) | 0.2527 | 0.5818 (0.1626–2.0819) | 0.4050 | 1.0994 (0.5535–2.1836) | 0.7866 |
| Age at inclusion | 26–35 | 1 | - | 1 | - | 1 | - | 1 | - |
|  | ≥36 | **0.35 (0.22–0.56)** | **<.0001** | **0.28 (0.12–0.64)** | **0.0025** | 0.38 (0.13–1.12) | 0.0780 | **0.31 (0.13–0.75)** | **0.0094** |
| Region | Europe | 1 | - | 1 | - | 1 | - | 1 | - |
|  | Asia Pacific | 1.23 (0.65–2.32) | 0.5334 | 1.27 (0.40–4.04) | 0.6887 | 1.60 (0.36–7.09) | 0.5349 | 1.06 (0.29–3.87) | 0.9273 |
|  | Latin America | 1.14 (0.59–2.22) | 0.6958 | 1.34 (0.41–4.40) | 0.6284 | 0.97 (0.18–5.34) | 0.9746 | 2.82 (0.79–10.10) | 0.1109 |
|  | North America | 1.11 (0.59–2.08) | 0.7395 | 2.04 (0.70–5.93) | 0.1894 | 1.95 (0.48–7.94) | 0.3537 | 2.45 (0.67–8.95) | 0.1766 |
| Age at first sexual intercourse grouped | ≥18 | 1 | - | 1 | - | 1 | - | 1 | - |
|  | 15-17 | 0.95 (0.59–1.53) | 0.8318 | 0.80 (0.37–1.75) | 0.5775 | 0.68 (0.23–2.01) | 0.4861 | 0.38 (0.14–1.05) | 0.0631 |
|  | <15 | 1.55 (0.73–3.30) | 0.2570 | 0.35 (0.04–2.75) | 0.3172 | Not estimated | - | 1.64 (0.41–6.57) | 0.4850 |
| Marital Status at baseline | Living or lived with partner | 1 | - | 1 | - | 1 | - | 1 | - |
|  | Single | **2.13 (1.22–3.70)** | **0.0077** | 1.99 (0.81–4.93) | 0.1347 | 2.83 (0.84–9.56) | 0.0935 | 1.62 (0.56–4.69) | 0.3707 |
| Smoking status at baseline | No | 1 | - | 1 | - | 1 | - | 1 | - |
|  | Yes | 0.88 (0.49–1.58) | 0.6743 | 1.33 (0.56–3.13) | 0.5205 | 1.44 (0.46–4.54) | 0.5285 | 0.83 (0.27–2.49) | 0.7338 |
| Number of sexual partners during the last year | 0 | 1 | - | 1 | - | 1 | - | 1 | - |
|  | 1 | 1.46 (0.65–3.26) | 0.3575 | 1.85 (0.42–8.13) | 0.4139 | 1.07 (0.22–5.14) | 0.9322 | 1.21 (0.34–4.30) | 0.7672 |
|  | ≥2 | **3.26 (1.35–7.83)** | **0.0084** | 4.64 (0.97–22.33) | 0.0554 | 2.83 (0.51–15.63) | 0.2338 | 1.40 (0.31– 6.39) | 0.6641 |
| Pregnancy | No | 1 | - | 1 | - | 1 | - | 1 | - |
|  | Yes | 1.17 (0.66–2.06) | 0.5985 | 1.14 (0.46–2.82) | 0.7749 | 1.03 (0.32–3.34) | 0.9624 | 1.02 (0.34–3.11) | 0.9704 |
| Chlamydia infection at baseline | No | 1 | - | 1 | - | 1 | - | 1 | - |
|  | Yes | 0.81 (0.34–1.95) | 0.6423 | 0.76 (0.17–3.32) | 0.7123 | 0.67 (0.08–5.28) | 0.7001 | 0.22 (0.02–2.12) | 0.1921 |
| History of HPV infection/treatment or not intact cervix | No | 1 | - | 1 | - | 1 | - | 1 | - |
|  | Yes | **1.70 (1.02–2.84)** | **0.0404** | 1.75 (0.77–3.98) | 0.1852 | 1.55 (0.49–4.92) | 0.4541 | **2.53 (1.09–5.90)** | **0.0316** |
| Previous cervical HPV-18 infection | No | - | - | - | - | - | - | 1 | - |
|  | Yes | - | - | - | - | - | - | **121.81 (53.39–277.91)** | **<.0001** |

HPV = human papillomavirus N = number of subjects used in the model; event = number of HPV-type specific 6-month persistent cervical infection; PI = persistent infection; CI = confidence interval. **Bold:** p-values < 0.05

**Table S5.** Multivariable Cox model for risk of newly detected human papillomavirus type 16 (HPV-16) infections or cervical abnormalities associated with HPV-16 including antibody level as time-dependent variable (per 10 EU units)

|  |  | **Antibody level per 10 units (time-dependent)** | | | | | | | |
| --- | --- | --- | --- | --- | --- | --- | --- | --- | --- |
|  |  | **Incident infection** | | **6-month PI** | | **12-month PI** | | **ASC-US+** | |
| Risk factor | Category | **Hazard ratio  (95% CI)** | **p-value** | **Hazard ratio  (95% CI)** | **p-value** | **Hazard ratio  (95% CI)** | **p-value** | **Hazard ratio  (95% CI)** | **p-value** |
| HPV-16 antibody level per 10 units as time-dependent |  | 1.0021 (0.9990–1.0053) | 0.1874 | 0.9958 (0.9698–1.0226) | 0.7580 | 0.9131 (0.7963–1.0470) | 0.1931 | 1.0018 (0.9937–1.0099) | 0.6683 |
| Age at inclusion | 26–35 | 1 | - | 1 | - | 1 | - | 1 | - |
|  | ≥36 | **0.58 (0.41–0.81)** | **0.0013** | 0.77 (0.49–1.22) | 0.2693 | 0.99 (0.54–1.81) | 0.9806 | 0.57 (0.31–1.05) | 0.0720 |
| Region | Europe | 1 | - | 1 | - | 1 | - | 1 | - |
|  | Asia Pacific | 1.22 (0.70–2.13) | 0.4813 | **2.73 (1.07–6.92)** | **0.0347** | 1.77 (0.61–5.10) | 0.2896 | 0.79 (0.29–2.15) | 0.6418 |
|  | Latin America | 1.60 (0.92–2.77) | 0.0960 | **4.05 (1.62–10.18)** | **0.0029** | 2.25 (0.79–6.46) | 0.1307 | 1.71 (0.68–4.33) | 0.2539 |
|  | North America | **2.36 (1.41–3.95)** | **0.0010** | **4.25 (1.73–10.46)** | **0.0016** | 1.93 (0.64–5.79) | 0.2429 | 1.05 (0.40–2.74) | 0.9164 |
| Age at first sexual intercourse grouped | ≥18 | 1 | - | 1 | - | 1 | - | 1 | - |
|  | 15-17 | 0.83 (0.57–1.20) | 0.3166 | 0.60 (0.34–1.04) | 0.0690 | 0.56 (0.26–1.22) | 0.1471 | 0.97 (0.49–1.91) | 0.9309 |
|  | <15 | 1.05 (0.55–2.03) | 0.8782 | 1.44 (0.66–3.17) | 0.3607 | 1.26 (0.41–3.81) | 0.6853 | 1.56 (0.56–4.33) | 0.3902 |
| Marital Status at baseline | Living or lived with partner | 1 | - | 1 | - | 1 | - | 1 | - |
|  | Single | 0.78 (0.48–1.27) | 0.3201 | 0.84 (0.43–1.65) | 0.6152 | 0.65 (0.22–1.92) | 0.4383 | 0.69 (0.28–1.72) | 0.4279 |
| Smoking status at baseline | No | 1 | - | 1 | - | 1 | - | 1 | - |
|  | Yes | 1.33 (0.87–2.04) | 0.1885 | 1.14 (0.60–2.14) | 0.6944 | 1.06 (0.43–2.59) | 0.8999 | 1.65 (0.81–3.38) | 0.1713 |
| Number of sexual partners during the last year | 0 | 1 | - | 1 | - | 1 | - | 1 | - |
|  | 1 | 0.88 (0.52–1.52) | 0.6569 | 1.02 (0.48–2.17) | 0.9679 | 2.26 (0.53–9.52) | 0.2684 | 0.86 (0.33–2.26) | 0.7592 |
|  | ≥2 | **2.37 (1.26–4.44)** | **0.0073** | **3.50 (1.46–8.39)** | **0.0050** | **8.40 (1.74–40.47)** | **0.0080** | 1.47 (0.47–4.57) | 0.5068 |
| Pregnancy | No | 1 | - | 1 | - | 1 | - | 1 | - |
|  | Yes | 0.73 (0.47–1.14) | 0.1671 | 0.55 (0.30–1.01) | 0.0543 | 0.80 (0.32–2.00) | 0.6314 | 0.88 (0.39–1.98) | 0.7607 |
| Chlamydia infection at baseline | No | 1 | - | 1 | - | 1 | - | 1 | - |
|  | Yes | 0.57 (0.26–1.26) | 0.1645 | 0.33 (0.08–1.41) | 0.1358 | 1.00 (0.22–4.56) | 0.9969 | 2.19 (0.61–7.89) | 0.2288 |
| History of HPV infection/treatment or not intact cervix | No | 1 | - | 1 | - | 1 | - | 1 | - |
|  | Yes | **1.53 (1.02–2.30)** | **0.0415** | 1.13 (0.60–2.11) | 0.7080 | 1.32 (0.58–3.03) | 0.5118 | 0.97 (0.42–2.24) | 0.9520 |
| Previous cervical HPV-16 infection | No | - | - | - | - | - | - | 1 | - |
|  | Yes | - | - | - | - | - | - | **117.09 (65.06–210.75)** | **<.0001** |

HPV = human papillomavirus N = number of subjects used in the model; event = number of HPV-type specific 6-month persistent

**Table S6.** Multivariable Cox model for risk of newly detected human papillomavirus type 18 (HPV-18) infections or cervical abnormalities associated with HPV-18 including antibody level as time-dependent variable (per 10 EU units)

|  |  | **Antibody level per 10 units (time-dependent)** | | | | | | | |
| --- | --- | --- | --- | --- | --- | --- | --- | --- | --- |
|  |  | **Incident infection** | | **6-month PI** | | **12-month PI** | | **ASC-US+** | |
| Risk factor | Category | **Hazard ratio  (95% CI)** | **p-value** | **Hazard ratio  (95% CI)** | **p-value** | **Hazard ratio  (95% CI)** | **p-value** | **Hazard ratio  (95% CI)** | **p-value** |
| HPV-18 antibody level per 10 units as time-dependent |  | 0.9965 (0.9548–1.0401) | 0.8721 | 0.9455 (0.8161–1.0954) | 0.4553 | 0.8597 (0.6100–1.2117) | 0.3880 | 1.0120 (0.9953 - 1.0289) | 0.1603 |
| Age at inclusion | 26–35 | 1 | - | 1 | - | 1 | - | 1 | - |
|  | ≥36 | **0.35 (0.22–0.56)** | **<.0001** | **0.28 (0.12–0.63)** | **0.0023** | 0.38 (0.13–1.11) | 0.0775 | **0.31 (0.13–0.75)** | **0.0099** |
| Region | Europe | 1 | - | 1 | - | 1 | - | 1 | - |
|  | Asia Pacific | 1.22 (0.64–2.31) | 0.5433 | 1.26 (0.40–4.02) | 0.6921 | 1.62 (0.36–7.18) | 0.5289 | 1.00 (0.28–3.63) | 0.9964 |
|  | Latin America | 1.14 (0.58–2.21) | 0.7064 | 1.35 (0.41–4.42) | 0.6215 | 0.98 (0.18–5.38) | 0.9804 | 2.80 (0.79–9.91) | 0.1098 |
|  | North America | 1.12 (0.60–2.09) | 0.7303 | 2.04 (0.70–5.91) | 0.1915 | 1.96 (0.48–8.01) | 0.3467 | 2.46 (0.68–8.90) | 0.1685 |
| Age at first sexual intercourse grouped | ≥18 | 1 | - | 1 | - | 1 | - | 1 | - |
|  | 15-17 | 0.95 (0.59–1.54) | 0.8476 | 0.78 (0.36–1.71) | 0.5388 | 0.68 (0.23–2.00) | 0.4834 | 0.40 (0.14–1.09) | 0.0735 |
|  | <15 | 1.57 (0.74–3.33) | 0.2451 | 0.35 (0.04–2.77) | 0.3204 | Not estimated | - | 1.61 (0.40–6.44) | 0.4974 |
| Marital Status at baseline | Living or lived with partner | 1 | - | 1 | - | 1 | - | 1 | - |
|  | Single | **2.13 (1.22–3.71)** | **0.0075** | 1.98 (0.80–4.88) | 0.1405 | 2.86 (0.85–9.62) | 0.0896 | 1.53 (0.53–4.46) | 0.4345 |
| Smoking status at baseline | No | 1 | - | 1 | - | 1 | - | 1 | - |
|  | Yes | 0.88 (0.49–1.58) | 0.6703 | 1.33 (0.56–3.14) | 0.5175 | 1.44 (0.46–4.53) | 0.5310 | 0.84 (0.28–2.53) | 0.7594 |
| Number of sexual partners during the last year | 0 | 1 | - | 1 | - | 1 | - | 1 | - |
|  | 1 | 1.46 (0.65–3.26) | 0.3563 | 1.85 (0.42–8.11) | 0.4165 | 1.09 (0.23–5.22) | 0.9178 | 1.17 (0.33–4.14) | 0.8097 |
|  | ≥2 | **3.27 (1.36–7.85)** | **0.0081** | 4.53 (0.94–21.76) | 0.0595 | 2.86 (0.52–15.81) | 0.2295 | 1.47 (0.33–6.64) | 0.6152 |
| Pregnancy | No | 1 | - | 1 | - | 1 | - | 1 | - |
|  | Yes | 1.17 (0.66–2.06) | 0.5932 | 1.13 (0.46–2.78) | 0.7941 | 1.01 (0.31–3.29) | 0.9829 | 1.08 (0.35–3.32) | 0.8975 |
| Chlamydia infection at baseline | No | 1 | - | 1 | - | 1 | - | 1 | - |
|  | Yes | 0.82 (0.34–1.96) | 0.6523 | 0.73 (0.17–3.19) | 0.6754 | 0.66 (0.08–5.22) | 0.6931 | 0.23 (0.03–2.13) | 0.1960 |
| History of HPV infection/treatment or not intact cervix | No | 1 | - | 1 | - | 1 | - | 1 | - |
|  | Yes | **1.71 (1.03–2.85)** | **0.0380** | 1.71 (0.75–3.90) | 0.2021 | 1.58 (0.50–4.99) | 0.4348 | **2.67 (1.14–6.25)** | **0.0232** |
| Previous cervical HPV-18 infection | No | - | - | - | - | - | - | 1 | - |
|  | Yes | - | - | - | - | - | - | **113.90 (49.31–263.09)** | **<.0001** |

HPV = human papillomavirus N = number of subjects used in the model; event = number of HPV-type specific 6-month persistent cervical infection; PI = persistent infection; CI = confidence interval. **Bold:** p-values < 0.05
